# Methanolic Extract of *Lysimachia Candida* Lindl. Prevents High-Fat High-Fructose-Induced Fatty Liver in Rats: Understanding the Molecular Mechanism Through Untargeted Metabolomics Study

**DOI:** 10.3389/fphar.2021.653872

**Published:** 2021-04-15

**Authors:** Parul Kamboj, Soumalya Sarkar, Sonu Kumar Gupta, Neema Bisht, Deepika Kumari, Md. Jahangir Alam, Sagar Barge, Bhaswati Kashyap, Barsha Deka, Simanta Bharadwaj, Seydur Rahman, Partha Pratim Dutta, Jagat C. Borah, Narayan Chandra Talukdar, Sanjay K. Banerjee, Yashwant Kumar

**Affiliations:** ^1^ Non-communicable Disease Group, Translational Health Science and Technology Institute (THSTI), Faridabad, India; ^2^ Department of Biotechnology, National Institute of Pharmaceutical Education and Research (NIPER), Guwahati, India; ^3^ Institute of Advanced Study in Science and Technology (IASST), Guwahati, India; ^4^ Assam Down Town University, Guwahati, India

**Keywords:** animal model, herbal extract, LC-MS/MS, bile acids, fatty acids, PPARα

## Abstract

Fatty liver is one of the most common metabolic syndrome affecting the global population. Presently, limited treatment modalities with symptomatic approach are available for alleviating fatty liver. Traditional and herbal treatment modalities have shown evidence to improve the disease pathology. In the present research work, evaluation of a selected medicinal plant *Lysimachia candida* Lindl. was carried out to investigate its beneficial effects on fatty liver disease in rats. Male Sprague Dawley (SD) rats were fed with high-fat high-fructose diet to induce fatty liver phenotypes. After induction for 15 weeks, methanolic extract of *Lysimachia candida* Lindl. (250 mg/kg b. w. p. o.) was administrated to the rats daily for the next 17 weeks. Blood samples were collected at different time points to analyze fasting blood glucose levels and relevant biochemical parameters important for the assessment of metabolic disease phenotypes. Liquid chromatography-mass spectrometry (LC-MS) based metabolomics was done to study the dynamics of metabolic changes in the serum during disease progression and how the medicinally important plant extract treatment reversed the metabolic diseases. Multivariate data analysis approaches have been employed to understand the metabolome changes and disease pathology. This study has identified the interplay of some metabolic pathways that alter the disease progression and their reversal after administration of the plant extract. Different group of metabolites mainly bile acids, fatty acids, carnitines, and their derivatives were found to be altered in the diseased rats. However, all the metabolites identified between control and disease groups are mainly related to lipid metabolism. The results depict that the treatment with the above-mentioned plant extract improves the regulation of aberrant lipid metabolism, and reverses the metabolic syndrome phenotype. Therefore, the present study reveals the potential mechanism of the herbal extract to prevent metabolic syndrome in rats.

## Introduction

The condition in which surplus lipids accumulate in the hepatocytes is known as fatty liver or hepatic steatosis (Allison, 2004). Accumulation of more than 5% lipids in hepatocytes is known as hepatic steatosis as the human liver does not have much capacity to store lipids (Li et al., 2018). Alcohol, chronic hepatitis C, Wilson’s disease, abetalipoproteinemia, and porphyria cutanea tarda are mostly found to be associated with hepatic steatosis. Medications like steroids, tamoxifen, and amiodarone are found to precipitate hepatic steatosis (Allison, 2004). Fatty liver disease can be categorized into two main types: non-alcoholic fatty liver disease (NAFLD) and alcoholic fatty liver disease (AFLD) (Singh et al., 2017). NAFLD is a chronic and progressive disease starting from non-alcoholic fatty liver (NAFL) to hepatic cirrhosis and ultimately leading to hepatocellular carcinoma (Sarkar et al., 2020). As per recent prevalence status, 20–46% of the world population has been stated to suffer from NAFLD (Li et al., 2018) with 15–40% patients in the western countries and 9–40% in Asian countries (Amarapurkar et al., 2007). There are no particular clinical symptoms of NAFLD other than discomfort in the right upper quadrant and fatigue. Some other signs include hepatomegaly, splenomegaly, spider naevi, and palmar erythema, although in most cases, the disease is asymptomatic with no signs of clinical manifestation (Allison, 2004).

NAFL is initiated by the deposition of small lipid droplets in hepatocytes, and overtime accumulation of these lipid droplets triggers lipotoxicity. The circulating cytokines together with elevated hepatic reactive oxygen species induce lobular inflammation, which results in the typical characteristic feature of non-alcoholic steatohepatitis (NASH) called hepatocellular ballooning. At advanced stages, the replacement of hepatic tissue with collagen results in the development of fibrotic scar tissues and leads to hepatic cirrhosis and ultimately hepatocellular carcinoma (Rinella, 2015; Carr et al., 2016; Foulds et al., 2017). The histopathological features of NAFLD are similar to alcoholic hepatic damage. Despite extensive research in understanding the pathophysiology of fatty liver diseases, there are still no targeted treatments available till date. The current treatment modalities for AFLD are identical to what it was 50 years ago i.e., abstinence, nutritional support, and corticosteroids (or pentoxifylline unless steroids are contraindicated). Whereas those for NAFLD include weight loss and co-morbidity management (Singh et al., 2017).

There are several evidences where traditional or herbal remedies are found to be useful in ameliorating several pathological features of fatty liver (Xiao et al., 2013). Based on the evidence to practice in North-east part of India by local people, we have selected a plant known as *Lysimachia candida* Lindl. belonging to the family *Primulaceae* (Yang et al., 2012)*.* The plant is native to the north-east region of India and mainly found in Assam and Manipur states. It is commonly known as loosestrife or kengoi in Manipuri. It is reported to have some pharmacological activity such as ailment of fever, swelling, fracture of bone, dermatitis, and antifungal activity (Xia et al., 2013). This plant has not been explored for its pharmacological effects on fatty liver disease. We have chosen this plant to explore its beneficial effect on fatty liver and validate this plant in the context of its therapeutic effects.

We have demonstrated that feeding high-fat high-fructose (HFHF) diet to Sprague Dawley (SD) rats over a period of 32 weeks not only induces fatty liver phenotypes such as biochemical and histopathological changes but also differentially regulates the level of several metabolites such as bile acids, unsaturated fatty acids and other metabolites involved in inflammation and homeostasis. Therefore, using the untargeted metabolomics approach, the metabolomic profile of rat serum from all three groups were used to identify the different metabolites involved in disease development and how the reversal of disease happens after the intervention of treatment. We identified various metabolites with the help of liquid chromatography-mass spectrometry (LC-MS) and both the enrichment and the pathway analyses demonstrated that peroxisome proliferator-activated receptor alpha is a central pathway through which *Lysimachia candida* Lindl. extract may show a beneficial effect on fatty liver.

## Materials and Methods

### Plant Collection and Identification of Bioactive Compounds

The whole plant *Lysamachia candida* Lindl. were collected from Moirang Kampu, Manipur (Latitude: 25°5’31.995”N to 24°39’45.25”N; Longitude: 94°8’49.324”E to 93°53’47.559”E) in June and July 2017. The Botanical Survey of India, Eastern Regional Centre, Shillong, Meghalaya authenticated the plant (Accession No. 95588). The *Lysimachia candida* Lindl. plant was air-dried for 2–3 weeks and then pulverized to a fine powder by the electric grinder. Exhaustive extraction was performed in a Soxhlet apparatus for 11 h using methanol (Merck) as a solvent. After which, the extract was concentrated under reduced vacuum pressure at 40°C in a rotary vacuum evaporator. The concentrated extracts were further lyophilized using Eyela Freeze Dryer (FDU-506, United States). 1 kg of fresh plant material yielded 100 g of dry powder losing 90% of moisture content and from extraction 1 kg plant material using methanol as solvent yielded 80 gm of dry extract. The final yield of methanolic extract was 8% w/w. Finally, the lyophilized extract was tested for residual methanol present using GC-MS and stored in a sterile container and placed at −20°C until further use. The stored lyophilized extract did not show the presence of any methanol. The extract was reconstituted in 100% methanol to identify bioactive compounds in LC-MS and LC-MSMS. The Orbitrap Fusion Mass Spectrometer was used to acquire data that is fitted with heated electrospray ionization (HESI) source. Both the negative and positive ions were scanned at 120,000 resolution in the MS1 mode and 30,000 resolution in the data-dependent MS2 scan mode. The spray voltage of 4,000 and 35,000 V were set for positive and negative modes, respectively. Auxiliary gas and Sheath gas were used and set to 11 and 42, respectively. The Mass scan analyzer was set to scan in the range of 50–1,000 m/z. For MS we have used an automatic gain control target of 200,000 ions with maximum injection time at 80 ms and 20,000 ions were used as an automatic gain control target at a maximum injection time of 60 ms for MSMS analysis. The methanolic extract was separated using HSS T3 column (100 × 2:1 mm i. d. 1.7 μm, Waters) in “UPLC Ultimate 3,000” and the temperature was maintained at 40°C. Mobile phase A containing water with 0.1% formic acid and acetonitrile with 0.1% formic acid was used in mobile phase B. The elution conditions were followed as 0 min, 1% B; 1 min, 15% B; 4 min, 35% B; 7 min, 95% B; 9 min, 95% B; 10 min, 1% B; and 14 min, 1% B with flow rate of 0.3 ml/min and 5 μl sample was injected. The data has been processed using Progenesis QI software with an untargeted approach. We have used different libraries of natural products i. e., spectral data matching with mzCloud and MassBank, Global Natural Products Social Molecular Networking (GNPS) for metabolite identification and in-house developed library of compounds based on purchase authentic metabolites standards and predicting the retention time of metabolites not available with us. Metabolites were confirmed through accurate mass, MS/MS fragmentation and retention time matching.

### Animals

To evaluate the potential effect of *Lysimachia candida* Lindl. on fatty liver, male Sprague Dawley (SD) rats were fed with high-fat high-fructose (HFHF) diet for 32 weeks. The animal protocols were approved by the Institutional Animal Ethics Committee (IAEC) of Translational Health Science and Technology Institute (THSTI), Faridabad, India (Protocol No. BIO-IAEC-3357). The study was performed in AAALAC accredited facility in Bioneeds Pvt. Ltd. Male SD rats aged between 8–12 weeks (200–250 g) was maintained at uniform laboratory conditions in standard steel cages and provided with food and water *ad libitum* during the study period. Rats were housed under standard laboratory conditions environmentally monitored, air-conditioned room with fresh air supply (12–15 air changes per hour), room temperature 22 ± 3°C and relative humidity 30–70%, with 12 h light and 12 h dark cycle. The temperature and relative humidity were recorded once daily.

### Study Design and Sample Collection

The plant extract was evaluated in rats using a curative model. Animals were randomly divided into three groups viz., control group, high-fat high-fructose (HFHF) group, and treatment group. All studies were carried out using six rats (*n* = 6) in each group. In the control group, the rats were fed with a normal chow diet for 32 weeks. At starting rats from the other two groups were fed with a high-fat high-fructose diet (Catalog no. D16030909, Research Diet, Inc. United States) for 15 weeks. After 15 weeks, the animals were selected and randomized into two groups viz., vehicle control (HFHF) and test group (Treatment), based on their fasting blood glucose level. The dose was selected based on efficacy study, LD50 was measured and it is found that dose is safe until 2.5 gm/kg oral dose. We chose one tenth of the dose as efficacy dose. 0.3% CMC vehicle and the plant extract (250 mg/kg/day) in the same vehicle were administered via oral gavage to the HFHF group and treatment group respectively, for 17 weeks along with HFHF diet. At the end of the study, the blood samples were collected in 2 ml ependroff tubes from the retro-orbital sinus for serum isolation. The animals were sacrificed and the liver was collected at the end of the study. All the serum samples and liver were stored at −80°C immediately for further use.

### Bodyweight and Food Intake

Changes in body weight and food intake patterns of rats in all groups were noted throughout the experimental period. The weight of each rat was recorded on day 0 and at weekly intervals throughout the study. The quantity of food consumed by each group was recorded weekly.

### Blood Glucose Level

Fasting blood glucose level was measured at day 0 (basal), before test item administration and thereafter once in 2 weeks throughout the experimental period. Animals were fasted overnight (approximately 12–16 h) before the experiment. Blood glucose level was measured using a Code-free glucometer through the tail prick method.

### Intraperitoneal Glucose Tolerance Test

Overnight fasted (12 h) rats from all groups were subjected to IPGTT at the end of study period. Freshly prepared glucose load of 2 g/kg of body weight was injected intraperitoneally (i.p.) just before 0 min of the experimental timepoint and a drop of blood was withdrawn from the tail vein by small puncture using a needle. Using a commercially available glucometer, blood glucose level was analyzed at 0, 15, 30, 60, and 120 min after injecting the glucose load.

### Biochemical Analysis

Serum and tissue triglycerides (TG) assay (Cayman Chemical, Triglyceride Colorimetric Assay Kit), free fatty acids (FFA) assay (FFA Kit, Biovision), and insulin assay (Insulin Kit, Cayman Chemical) were performed according to the instructions provided by the manufacture. For the hepatic TG, liver tissues of each rat were homogenized in NP-40 lysis buffer and centrifuged at 12,000 g for 30 min at 4°C.

### Histopathology

At the end of the experimental period, each animal was sacrificed and tissues were collected and washed by buffered normal saline. Tissues were fixed into 10% formalin solution and then dehydrated through graded alcohol series (70–100%), cleared in xylene, and embedded in paraffin. 5–6 µm thick paraffin sections were cut and stained with hematoxylin-eosin (H&E) and Masson’s trichome (MT). Cryopreserved samples were used for Oil-O-Red (OOR) staining. These slides were investigated and analyzed under a light microscope at 40X and 60X magnification.

### Metabolomics Analysis

#### Metabolomics Sample Preparation

Prior to experimentation, the serum samples were thawed at 4°C and kept for 30 min(s). Then, metabolite extraction was carried out by adding 150 µl of ice-cold 100% methanol (LCMS-grade, Merck) in 50 µl serum. The samples were briefly vortexed ∼30 s and left on ice for 20 min(s) protein precipitation. Then, samples were centrifuged at 10,000 rpm for 10 min at 4°C. Approximately 150 µl of the supernatant was collected and divided into two microcentrifuge tubes and evaporated to dryness in a vacuum dryer. The samples were stored at −80°C until data acquisition. For reverse-phase chromatography, samples were resuspended in 100 μl of water: methanol (15:85, V/V) and for polar phase chromatography samples were resuspended in 100 μl of water: acetonitrile (50:50, V/V). After brief vortexing, the mixture was centrifuged at 10,000 rpm for 10 min at 4°C. All the supernatants were collected in fresh vials. A Quality Control (QC) sample was prepared by pooling 10 µl from each vial in a microcentrifuge tube (Naz et al., 2017).

#### Metabolite Measurement

Sample acquisition was done on Dionex Ultimate 3000 RS LC (Thermo Scientific) coupled with Orbitrap fusion MS (Thermo Scientific). Electrospray ionization was used for the better coverage and identification of polar and nonpolar metabolites. Metabolites were separated on reverse phase (C18) and hydrophilic interaction chromatography (HILIC column) in separate runs. The data was acquired in positive and negative ionization modes. The reverse phase column was HSS T3 and the HILIC column was XBridge BEH Amide (Waters Corporation). For, polar compound separation, solvent A was 20 mM ammonium acetate in the water of PH 9.0, and mobile phase B was 100% acetonitrile. The elution gradient starts from 85% B to 10% B over 14 min with a flow rate of 0.35 ml/min. For the reverse-phase, Solvent A was water and B was methanol with 0.1% formic acids added in both. The elution gradient starts with 1% B to 95% B over 10 min with a flow rate of 0.3 ml/min (Yuan et al., 2012; Kumar et al., 2020).

For each sample, injection volume was 5 µl and column temperature was kept at 40°C. The MS1 mass scan range was set to 65–1,000 da. The resolution for MS was 120,000 and for MSMS was 30,000. The MSMS analysis was acquired in ddMS2 mode. The flow rate for Auxiliary and Sheath gas was set at 42 arbs and 11 arbs, respectively. The spray voltage was 3.5 kV for positive mode and 3.0 kV for negative mode. The temperature of the vaporizer was 310°C and the capillary temperature was 300°C. Pool quality control (QC) sample was run after every five samples to monitor signal variation and drift in mass error.

### Data Analysis

Metabolomic data analysis was done using compound discoverer (Thermo Scientific) 3.0. Retention time alignment, peak picking, and database search were done in compound discoverer 3.0 with default settings. Metabolite identification was done using the in-house library with retention time, accurate mass, and fragmentation pattern. Additionally, we used spectral library downloaded from different online databases. All features whose fragmentation score > 30% and retention time were within ± 0.5 min of the in-house library and a CV score of < 30% in the QC samples were selected for further statistical analysis. The statistical analysis and pathway enrichment analysis were done on freely available online metabolomics data analysis tool, MetaboAnalyst (https://www.metaboanalyst.ca/). One-way analysis of variance (ANOVA) and Bonferroni’s test was applied to compare values between control and treated groups using Graph Pad Prism. Results were expressed as mean ± standard deviation (SD). The values depicting *p* < 0.05 were considered as statistically significant.

## Results

### Characterization of Methanolic Extract of *Lysimachia Candida* Lindl. by Liquid Chromatography-Mass Spectrometry

The methanolic extract of *Lysimachia candida* Lindl. was characterised by LC-ESI-MS/MS. The LC-MS/MS chromatographic profile (both negetaive mode and positive mode) showed the presence of several metabolites which include *trans*-3-indoleacetic acid, l-phenylalanine, N-methylalanine, dehydrophyto sphingosine, 3-phenyllactic acid, azelate, 3,5 dimethoxycinnamic acid and cinnamic acid (Supplementary Figures S1, S2). All these compounds were identified by MS/MS fragmentation pattern (Supplementary Figure S3A–I).

### Effect of Methanolic Extract of *Lysimachia Candida* Lindl. on Body Weight of High-Fat High-Fructose Diet-Fed Rats

**FIGURE 1:**
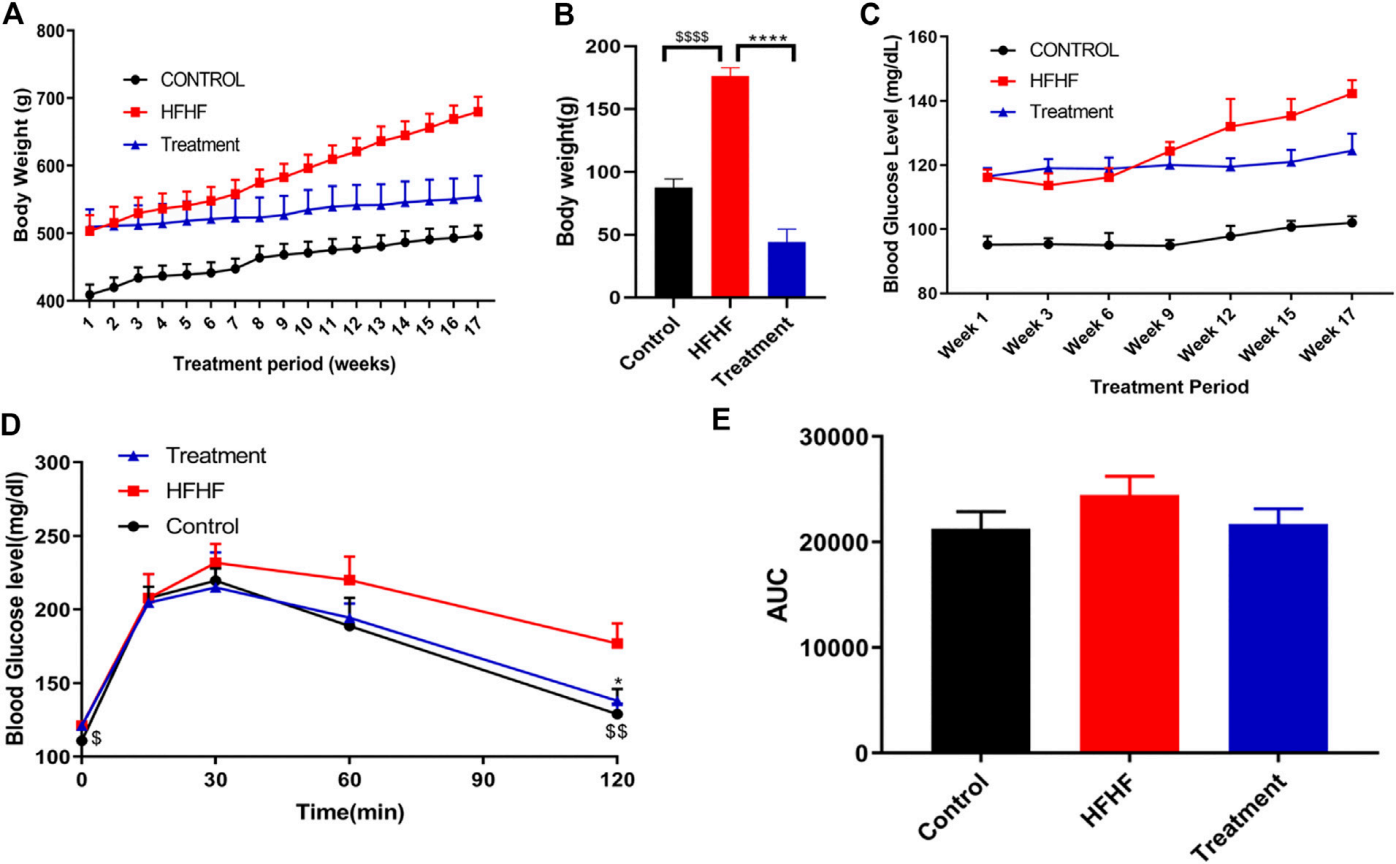
Effect of *Lysimachia candida* Lindl. extract administration on the physical parameters of rats **(A)** Body weight, **(B)** Body weight change, **(C)** Fasting blood glucose, **(D)** IPGTT, **(E)** AUC of glucose level. Data are expressed as mean ± SD (*n* = 6) each group, *p* < 0.0001 **** signifies the statistical difference between HFHF and Treatment, *p* < 0.05 * signifies the statistical difference between HFHF and Treatment, *p* < 0.0001 $$$$ signifies the statistical difference between HFHF and Control, *p* < 0.005 $$ signifies the statistical difference between HFHF and Control, *p* < 0.05 $ signifies the statistical difference between HFHF and Control.

During the experimental period, increase in body weight was observed in all the groups. A substantial increase (*p* < 0.001) in body weight was observed in HFHF diet-fed animals **(** Figures 1A,B **)** when compared to normal chow diet animals (Control). Significant (*p* < 0.001) reduction of body weight gain was observed in HFHF diet-fed animals treated with a dose of 250 mg/kg p. o. methanolic extract of *Lysimachia candida* Lindl. over a period of 17 weeks **(** Figures 1A,B **)**.

### Effect of Methanolic Extract of *Lysimachia Candida* Lindl. on Fasting Blood Glucose Level and Insulin Resistance in the High-Fat High-Fructose Diet-Fed Rats

The study showed that there was an increase in fasting blood glucose levels after 15 weeks of HFHF diet. Fasting blood glucose levels were further increased after 17 weeks of HFHF diet (Figure 1C). Oral administration of methanolic extract of *Lysimachia candida* Lindl. to the HFHF-fed animals for 17 weeks showed alleviation in the fasting blood glucose levels (Figure 1C).

Intraperitoneal glucose tolerance test (IPGTT) was carried out to check insulin resistance of the different experimental groups. As compared to the HFHF group, administration of methanolic extract of *Lysimachia candida* Lindl. prevented the rise in serum glucose levels after 17 weeks of treatment (Figure 1D). Similarly, the area under the curve (AUC) of glucose level in the IPGTT also reveals that HFHF groups have a higher area than control whereas treatment with the extract reduced the AUC to that of the Control group (Figure 1E).

### Effect of Methanolic Extract of *Lysimachia Candida* Lindl. on Biochemical Parameters of the High-Fat High-Fructose Diet-Fed Rats

**FIGURE 2:**
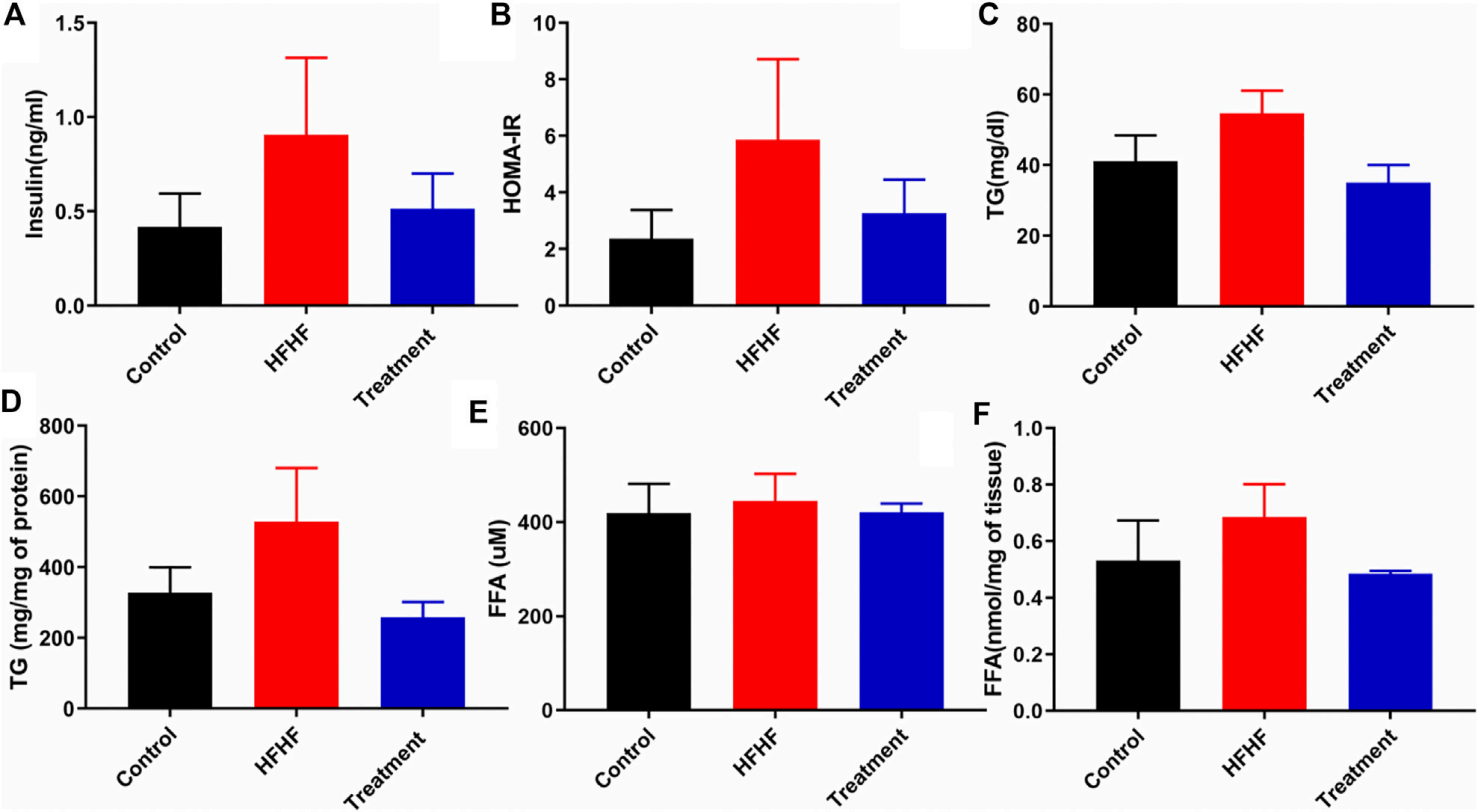
Effect of *Lysimachia candida* Lindl. extract administration on the serum and liver levels of biochemical parameters of rats **(A)** Serum insulin, **(B)** HOMA IR, **(C)** Serum triglycerides, **(D)** Liver triglycerides, **(E)** Serum free fatty acid, **(F)** Liver free fatty acid. Data are expressed as mean ± SD (*n* = 6) each group, compared with HFHF group.

To further explore the effectiveness of the *Lysimachia candida* Lindl. extract, biochemical parameters in the HFHF diet-fed rats were monitored. As shown in Figure 2A, serum insulin level was markedly increased in the HFHF group compared to the Control and treatment groups at the end of the study. Similarly, the HOMA IR data confirmed that there was remarkable insulin resistance in the HFHF group when compared to the other two groups. The increased levels of serum insulin and HOMA IR were decreased in the treatment group when compared to the HFHF group (Figures 2A,B). The increased level of serum and hepatic triglycerides (TG) in the HFHF group was decreased to the baseline in the treatment group **(** Figures 2C,D). However, there was no change in the serum of FFA levels among the three groups (Figure 2E). Similar to the change in TG level, increased hepatic FFA was observed in the HFHF group and it decreased after treatment (Figure 2F). Taken together, these data showed that the biochemical parameters were remarkably recovered to baseline in the treatment group although data were not statistically significant.

### Effect of Methanolic Extract of *Lysimachia Candida* Lindl. on Histological Changes in High-Fat High-Fructose Diet-Fed Rats

**FIGURE 3:**
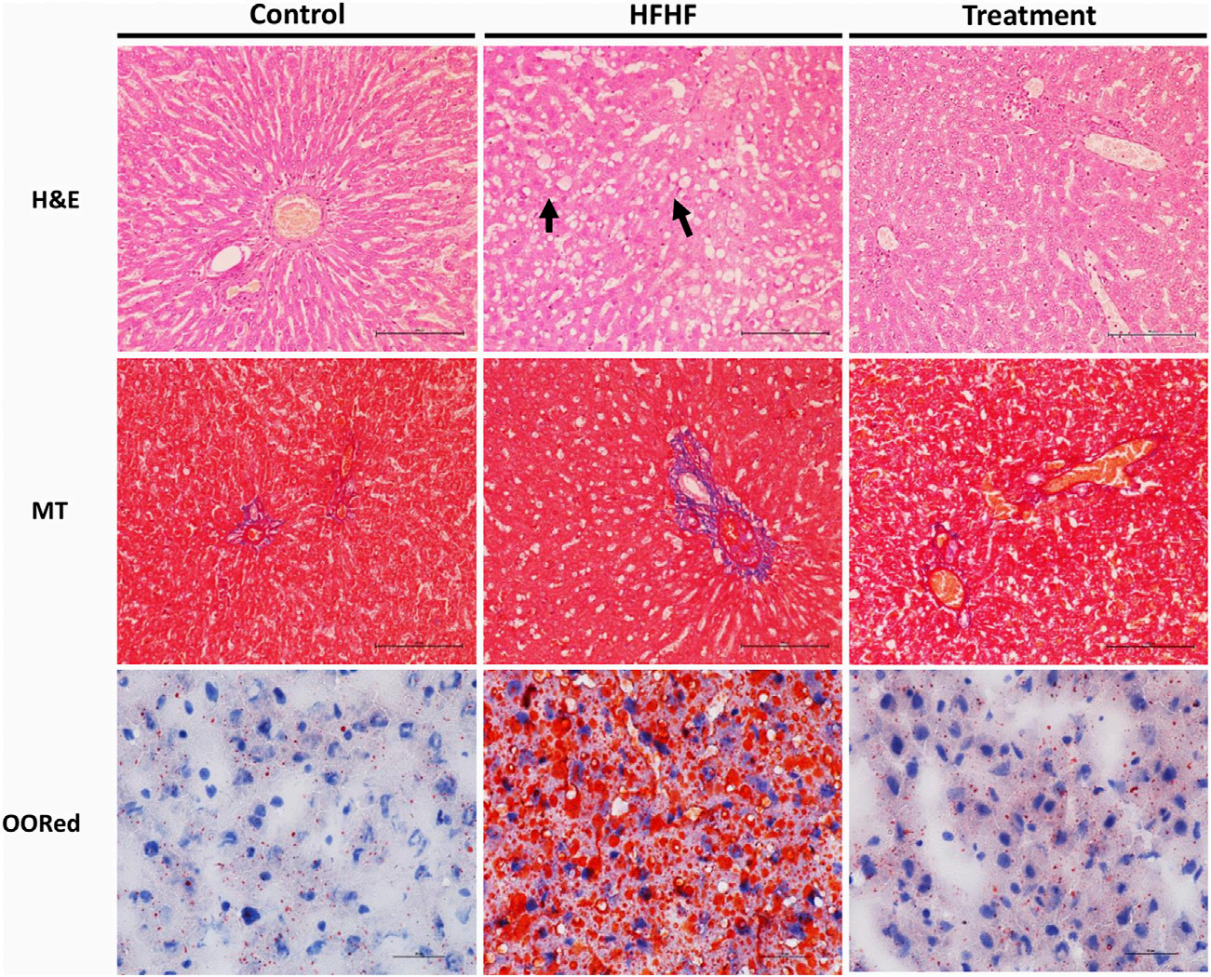
Representative sections of rat liver showing histological alteration in the liver by H&E stain (40X) arrows indicating fat globules, MT stain (40X) indicating fibrosis, and Oil-O-Red stain (60X) indicating the fat accumulation.

To examine the histological changes in all the groups, the slides were stained with H&E, Masson’s Trichrome stain and Oil-O-Red. H&E-stained liver sections of the control group revealed the normal hepatic structure and no hepatocellular ballooning and degeneration characterized by cell swelling with empty intracellular content, indicating cell necrosis and inflammation. By contrast, the liver histology of the HFHF group showed discernible changes such as increased fat accumulation, as well as ballooning and degeneration of hepatocytes. Interestingly, the liver sections of the treatment group showed no to mild ballooning and degeneration of hepatocytes. It seems to be in the recovery stage as the central vein was found to be slightly dilated but cells were healthy (Figure 3). Masson’s Trichrome stained sections did not show much changes in collagen fiber pattern except a little thinning of connective tissue around the portal tracts of HFHF diet-fed animals (HFHF) as compared to control. Interestingly, treatment group animals did not show any such thinning effect (Figure 3). Oil-O-Red staining unveiled huge macrovascular lipid accumulation in all areas of the lobe indicating steatohepatitis in the HFHF group when compared to the Control and Treatment groups. However, *Lysimachia candida* Lindl. extract treatment reduced the lipid droplets to the levels observed in the control group (Figure 3).

### Effect of Methanolic Extract of *Lysimachia Candida* Lindl. on Metabolite Profiling of High-Fat High-Fructose Diet-Fed Rat Serum

**FIGURE 4:**
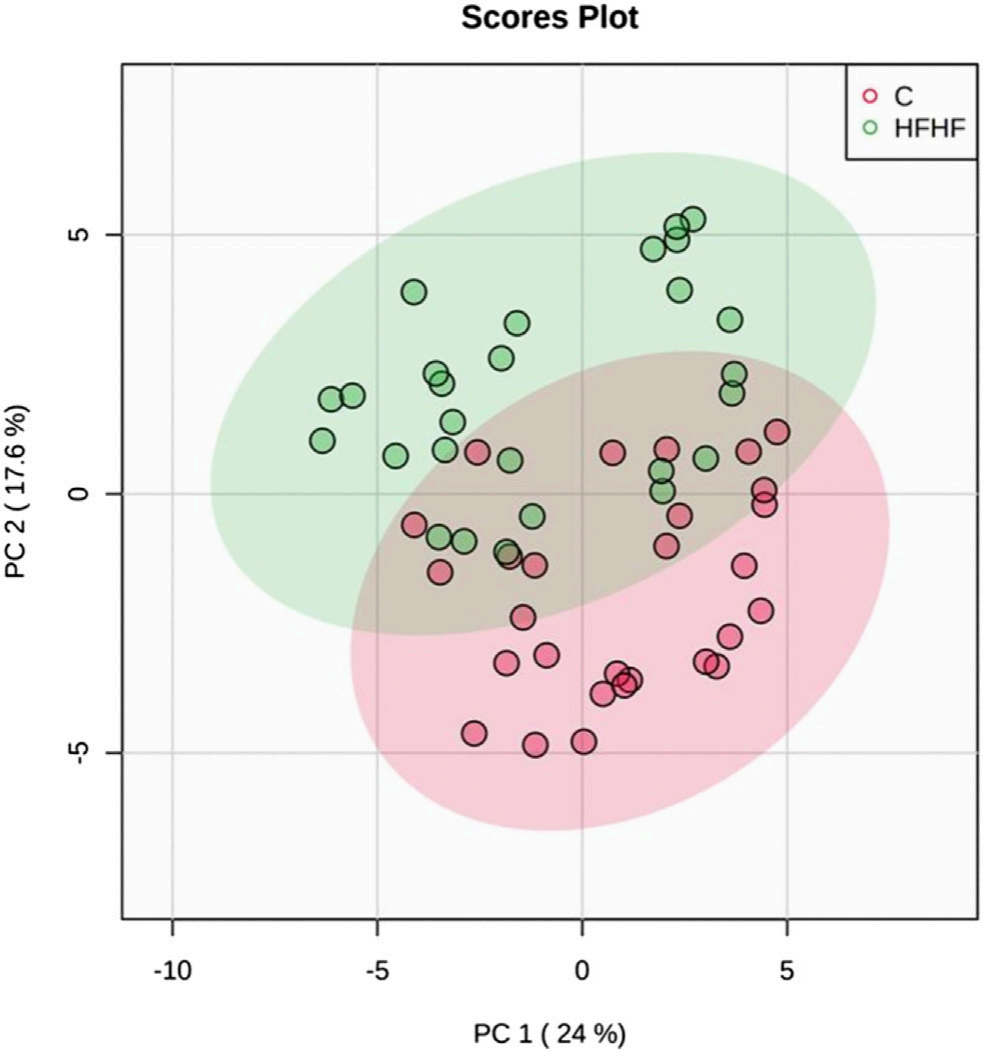
Metabolomic analysis of HFHF induced fatty liver. Principal component analysis (PCA) of control and high-fat high-fructose fed rat at different time points.

**FIGURE 5:**
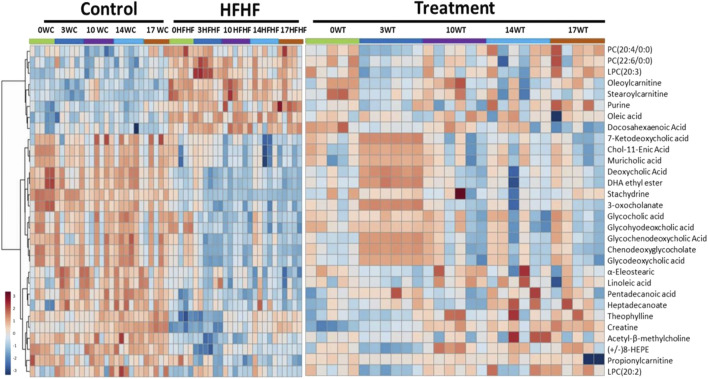
Metabolomic analysis of HFHF-induced fatty liver and the effect of the treatment (*Lysimachia candida* Lindl.). Heat map analysis of different metabolites depicting the effect of treatment on the altered metabolites. Hot (red) colour indicates high exprerssion and cool (blue) colour represents low expression.

**FIGURE 6:**
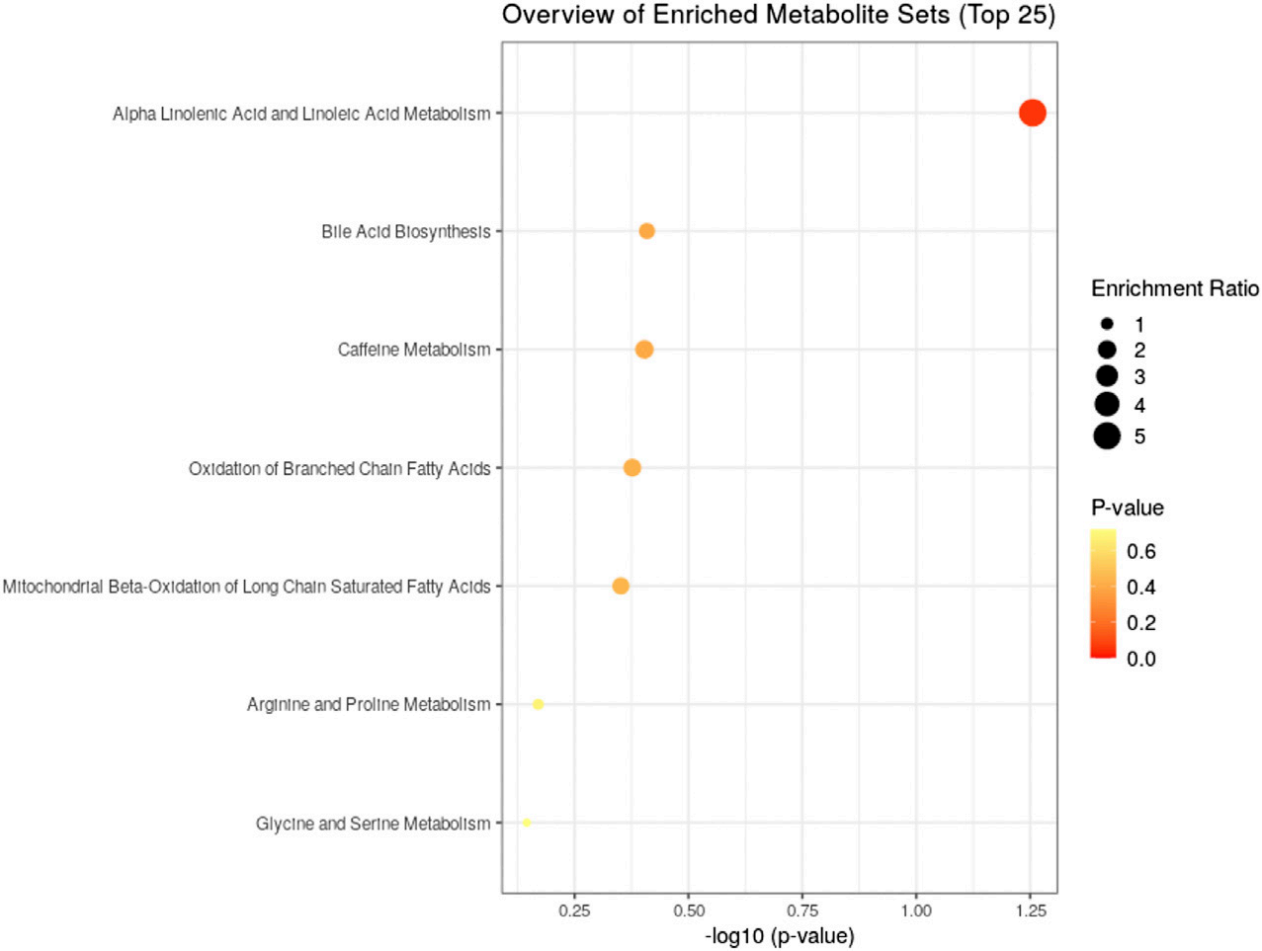
Identified pathway and the metabolites which have the most impact on the pathway using MetaboAnalyst from enriched metabolites.

To understand the mechanism by which methanolic extract of *Lysimachia candida* Lindl. treatment reverses the disease pathophysiology in high-fat high-fructose (HFHF)-fed rats, we conducted untargeted metabolic profiling using orbitrap fusion mass spectrometry (MS) coupled with ultra-performance liquid chromatography (UPLC). To maximize the coverage of different classes of metabolites and to identify the different classes of metabolites, we have run the rat serum on reverse phase and hydrophilic column. We have identified a total of 155 metabolites in serum samples after combining data from the positive and negative modes (Supplementary Table S1). Principal component analysis (PCA) has clearly indicated a good separation between the control and HFHF rat serum metabolome. As shown in Figure 4, principal component 1 explains 24% of data and principal component 2 explains 17.6% of the data. The third component explains another 10.9% of the data, then fourth and fifth components explain 7.2 and 4.6% of data respectively. Total variation explained by all the five components is 64.3%. Further, we did a clustering analysis of data to visualize the different metabolites in more detail. Comparison among control, HFHF, and treatment have been done by ANOVA and significantly altered metabolites have been shown in the heatmap (Figure 5). Comparison between control and HFHF rat serum metabolites using clustering indicated the change in bile acids, biosynthesis of unsaturated fatty acid, carnitines, and other metabolites involved in the peroxisome proliferator-activated receptors (PPAR) pathways (Figure 5). Interestingly, the expression of these metabolites has been found to be reversed in the Treatment group. The expression of metabolites such as (+/-)8-hydroxy eicosapentaenoic acid ((+/-)8-HEPE), pentadecanoic acid, and heptadecanoate was decreased in HFHF group and reversed after the treatment with the plant extract. Similarly, different bile acid levels such as muricholic acid, deoxycholic acid, glycocholic acid, glycochenodeoxycholic acid, and glycodeoxycholic acid were decreased in the serum of HFHF group rats and were found to be normalized after plant extract treatment in early time points (Figure 5). We have also observed that unsaturated fatty acids like linoleic acid and *α*-eleostearic were decreased in the HFHF group and reversed after treatment (Figure 5). Pathway analysis using metaboanalyst using different metabolites showed that important pathway intervene were linoleic acid metabolism, biosynthesis of unsaturated fatty acids, glycerophospholipid metabolism, and primary bile acid biosynthesis pathway (Figure 6).

## Discussion

Non-alcoholic fatty liver disease (NAFLD) is increasingly recognized as a major health problem in developed and developing countries. It includes a spectrum of liver disease ranging from simple steatosis to non-alcoholic steatohepatitis (NASH), advanced fibrosis, and rarely, progression to cirrhosis. NAFLD is associated with several diseases like obesity, insulin resistance, type 2 diabetes mellitus, hyperlipidemia, hypertension, and metabolic syndrome (Bedogni et al., 2005). It has been shown that NAFLD is strongly associated with the features of metabolic syndrome. Insulin resistance is a key pathogenic factor in both NAFLD and metabolic syndrome. Data from experimental and clinical studies indicate that NAFLD may be the hepatic manifestation of metabolic syndrome (Marchesini et al., 2001). There are no drugs i.e., small molecules approved by the FDA to treat NAFLD or NASH. However, few pharmacological modalities have been practiced to ease the associated co-morbidities. Considering the presence of several active biomolecules in herbs and their well-proven role in health and disease, further research is necessary to confirm the therapeutic role of herbs in patient with NAFLD. Few herbal preparations have already found to be beneficial in ameliorating several pathological features of fatty liver (Hong et al., 2006; Park et al., 2006; Bose et al., 2008; Chidambaram et al., 2010). Here, in the present study, we have selected the plant *Lysimachia candida* Lindl. widely used by the native population of the northeastern region to cure different metabolic disease, to explore its potential role in preventing HFHF-induced fatty liver in SD rats.

Previously, we and others showed that chronic feeding of high-fat high-fructose diet causes increase in body weight, insulin resistance, and hyperglycemia (Nizami et al., 2019). In the present study, we have observed a remarkable increase in body weight in the HFHF group indicating obesity in rats. However, reduction in body weight was observed in high-fat high-fructose diet-fed rats after treatment with methanolic extract of *Lysimachia candida*, with not much change in its timeline. Similarly, fasting blood glucose (FBG) level was found to be decreased in the treatment group. The data clearly demonstrate that the methanolic extract of *Lysimachia candida* Lindl. was able to control the FBG levels in the treatment group. The IPGTT curve and its area under the curve reveals that there is an increase in insulin resistance in HFHF group. High levels of insulin were also observed in the HFHF group contrary to the Control group. Insulin resistance in the HFHF was confirmed by the increase in the HOMA IR whereas the treatment with methanolic extract of *Lysimachia candida* Lindl. alleviated the HOMA IR near to the Control, suggesting its potential activity to combat insulin resistance. To check the lipid levels, we measured the TG and FFAs in both serum and hepatic tissue. We found that the serum and hepatic triglyceride levels were increased in the HFHF group while TG levels decreased in the Treatment group when compared with Control. Hence, again the methanolic extract of *Lysimachia candida* Lindl. was successfully able to normalize the TG levels in both serum and liver. Similarly, high levels of hepatic FFAs were observed in the HFHF group, which was decreased after treatment. Therefore, the methanolic extract of *Lysimachia candida* Lindl. is capable of reversing and managing body weight changes, FBG, insulin resistance, TG, and FFA levels.

Histology analysis confirmed the development of fatty liver with early fibrosis. Since Oil-O-Red specifically stains triglycerides together with cholesterol ester, this stain represents the real representation of lipid accumulation (Sarkar et al., 2020). We have observed a high level of lipid deposition in the HFHF group. However, the methanolic extract of *Lysimachia candida* Lindl. administration reduced the fat accumulation similar to the control group. Liver samples from the HFHF group exhibited prominent steatosis, ballooning, and lobular inflammation with mild fibrosis, while the Treatment group unveiled reduced levels of steatohepatitis with no fibrosis. Overall, the histopathology study confirmed the presence of hepatic steatosis, ballooning, lobular inflammation, and perisinusoidal fibrosis in the disease group, which was alleviated in the treatment group.

After confirming the fatty liver phenotype, we wanted to correlate the phenotypic changes in HFHF rats with serum metabolic profiles. The untargeted metabolomic analysis with multivariate data analysis (Principal component analysis) and clustering revealed a clear separation of rat serum metabolome between Control and HFHF groups. Principal component analysis clearly indicates the difference in metabolome in HFHF as compared to control at different time points. Clustering analysis also indicates change in levels of different metabolites in the control and HFHF group. Pathway enrichment analysis of different metabolites suggests a change in linoleic acid metabolism, biosynthesis of unsaturated fatty acids, glycerophospholipid metabolism, and primary bile acid biosynthesis pathway. Hence, we could infer from our result that high-fat high-fructose diet has altered various metabolites from the normal physiological level. Changes in bile acids, unsaturated fatty acids, carnitines, and other metabolites involved in the peroxisome proliferator-activated receptors (PPAR) pathways were found in HFHF animals. Interestingly, the altered levels of these metabolites have been found to be reversed in the treatment group. PPAR alpha and PPAR gamma are highly expressed in the liver and play a significant role in the synthesis of bile acid and fatty acid uptake (Lee et al., 1995; Mandard et al., 2004). Oxidation of fatty acid and expression of fatty acid transport protein shares a close relationship with nuclear receptor PPARα and PPARγ in liver steatosis (Pyper et al., 2010).

The serum metabolites such as (+/-)8-hydroxyeicosapentaenoic acid ((+/-)8-HEPE)), pentadecanoic acid, heptadecanoate were downregulated in the HFHF group and interestingly, plant extract treatment has reversed their expression. (+/-) 8-hydroxyeicosapentaenoic acid acts as a ligand of PPAR (Forman et al., 1997; Yamada et al., 2014). For the first time, Yamada et al. demonstrated that orally administered 8-HEPE can activate PPARα, resulting in the decrease of plasma and hepatic TG levels (Yamada et al., 2016). As stated, 8-HEPE is a stimulator of PPARα, hence decrease levels of 8-HEPE can reduce the activation of PPARα and increase the serum and hepatic TG levels as we observed in the HFHF group. However, the methanolic extract of *Lysimachia candida* Lindl. administration increased the serum 8-HEPE levels similar to the control group. Two fatty acids, pentadecanoic acid (C15) and heptadecanoic acid (C17) were reduced in HFHF group. Epidemiological researches have revealed that plasma concentrations of odd chain saturated fatty acids like pentadecanoic acid and heptadecanoic acid are related to reduced risks for metabolic disorders like type II diabetes and coronary heart disease (Jenkins et al., 2015). Interestingly, the treatment with methanolic extract of *Lysimachia candida* Lindl. was able to upregulate these odd chain saturated fatty acids levels similar to Control.

**FIGURE 7:**
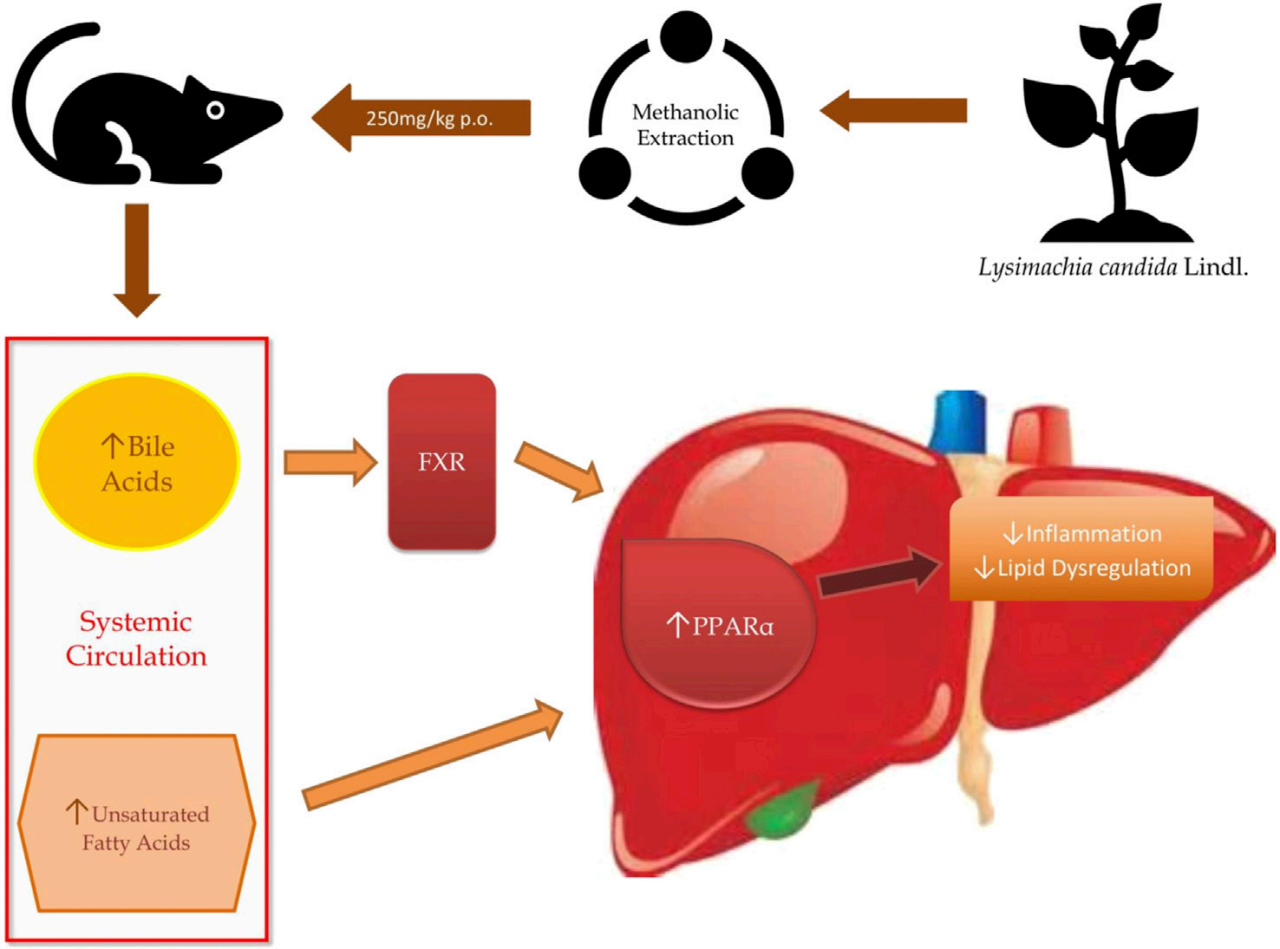
Schematic diagram representing the results and possible pathway unveiled by our study.

Bile acids play a crucial role in maintaining the level of both free and conjugated forms of bile acids in the peripheral circulation. In hepatic disease, levels of bile acids are aberrated especially in synthesis, excretion, and reabsorption of bile acids, which raises the level of total bile acids. Some studies have demonstrated the effect of liver injury on bile acid metabolism (Wang et al., 2012; Beyoğlu and Idle, 2013). A previous study reported that the levels of bile acid were found to be low in carbon tetrachloride (CCl4) induced fatty liver rats (Zhang et al., 2019). When there is a disruption in the bile acid metabolism, it leads to a decrease in the activation of nuclear receptors such as FXR (farnesoid X receptor) and dysregulation in these receptors leads to NAFLD (Parséus et al., 2017; Chen et al., 2019; Liu et al., 2020). Chenodeoxycholic acid, muricholic acid and other cholic acids are primary bile acids produced in the liver as derivatives of cholesterol and secreted in the intestine as taurine and glycine conjugates. These bile acids interact with FXR and regulate glycemia and other metabolic functions (Grabherr et al., 2019). In the present study, we have found that different bile acids such as Muricholic acid, Deoxycholic Acid, Glycocholic acid, Glycochenodeoxycholic Acid, and Glycodeoxycholic acid were lowered in the serum of HFHF rats. However, their serum levels were normalized after the treatment of the methanolic extract of *Lysimachia candida* Lindl. especially after 3 weeks. Lower serum levels of bile acids in the HFHF group may reduce the activation of FXR and enhance the fat accumulation in the rat liver which is similar to NAFLD pathologies. Our findings might be working through similar pathway as evidenced in the study by Sun (Sun et al., 2018). Bile acids activate mRNA expression of PPARα via binding of FXR in the PPAR promoter (Pineda Torra et al., 2003). Therefore, it seems that the FXR-PPARα pathway might be involved for the attenuation of fatty liver after the treatment of methanolic extract of *Lysimachia candida* Lindl (Figure 7)*.*

Unsaturated fatty acids like Linoleic acid and *α*-Eleostearic acid were lowered in HFHF rats and increased after treatment of the plant extract. Our finding was similar to previous study where it was shown that Linoleic acid, an essential polyunsaturated fatty acid, was declined in non-alcoholic steatohepatitis (NASH) (Puri et al., 2009). *α*-Eleostearic acid, a PPARα activator, is lowered in HFHF rats. *α*-Eleostearic acid also upregulates peroxisomal acyl-CoA oxidase activity and cytochrome P450 4A1 genes in rats, which in turn activates PPARα (Chao et al., 2001). Hence, lower levels of *α*-Eleostearic acid in HFHF group may cause inactivation of PPARα pathways, and therefore fatty liver pathologies. The methanolic extract of *Lysimachia candida* Lindl. was able to nullify these pathological features by upregulating *α*-Eleostearic acid expression and activating PPARα pathways (Figure 7).

Pathway analysis, using metaboanalyst by the exploration of different metabolites confirms that important distrupted pathway are linoleic acid metabolism, biosynthesis of unsaturated fatty acids, glycerophospholipid metabolism, and primary bile acid biosynthesis pathway (Figure 6). The summarized pathway of our study is revealed in (Figure 7).

Although we have established the link between altered serum metabolites levels and fatty liver phenotypes, there are some limitations of the study. First, there are many different components in the methanolic extract of *Lysimachia candida* Lindl. and we have not looked which particular component(s) or compound(s) is activating PPARα. Secondly, the molecular signaling pathway of PPARα activation has not been deciphered after the treatment with plant extract.

## Conclusion

Our study concludes that the methanolic extract of *Lysimachia candida* Lindl. reduces insulin resistance along with fatty liver phenotypes in rats. After analyzing various metabolites and pathways associated with fatty liver, we found that activation of PPARα by methanolic extract of *Lysimachia candida* Lindl. might be responsible to prevent the high-fat high-fructose induced fatty liver in rats.

## Data Availability

The authors acknowledge that the data presented in this study must be deposited and made publicly available in an acceptable repository, prior to publication. Frontiers cannot accept a article that does not adhere to our open data policies.
