# Supplementary figures and images for: Methanolic Extract of Lysimachia Candida Lindl. Prevents High-Fat High-Fructose-Induced Fatty Liver in Rats: Understanding the Molecular Mechanism Through Untargeted Metabolomics Study
Source: Front Pharmacol. 2021 Apr 15;12:653872. doi: 10.3389/fphar.2021.653872 (PMC8082144; doi:10.3389/fphar.2021.653872)

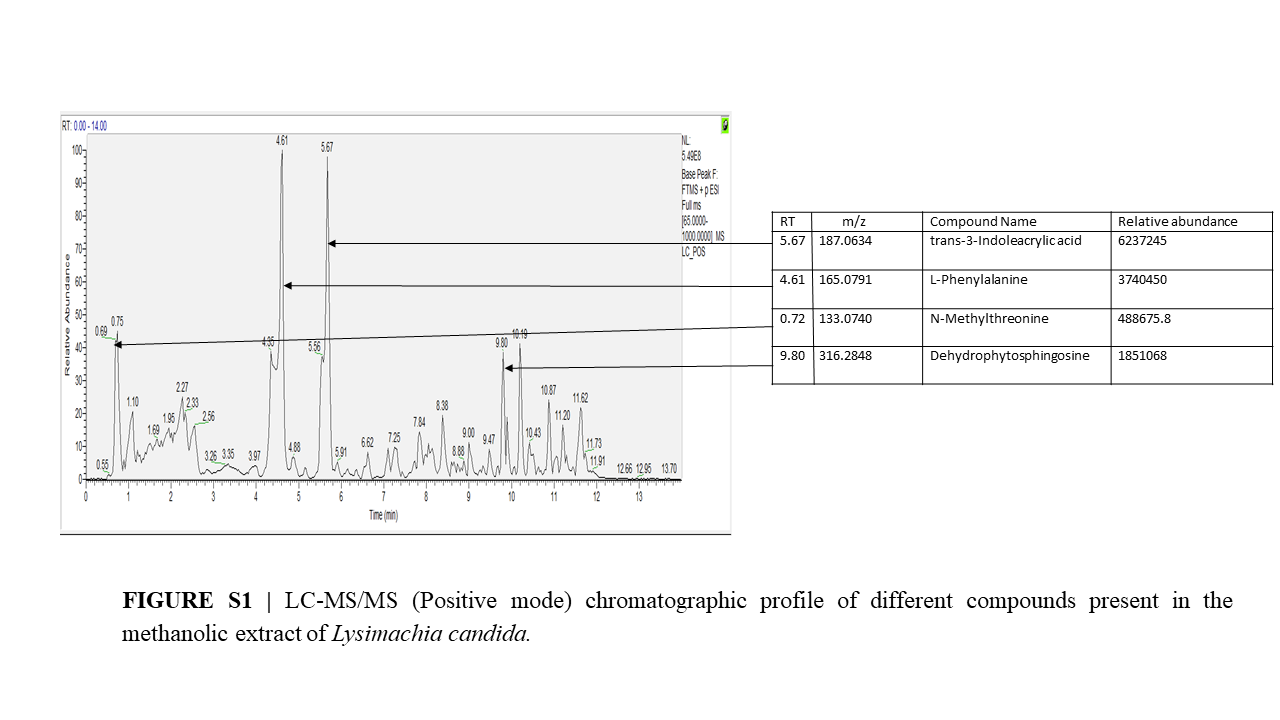

Supplement: Supplementary file 1 [file image1.tif]

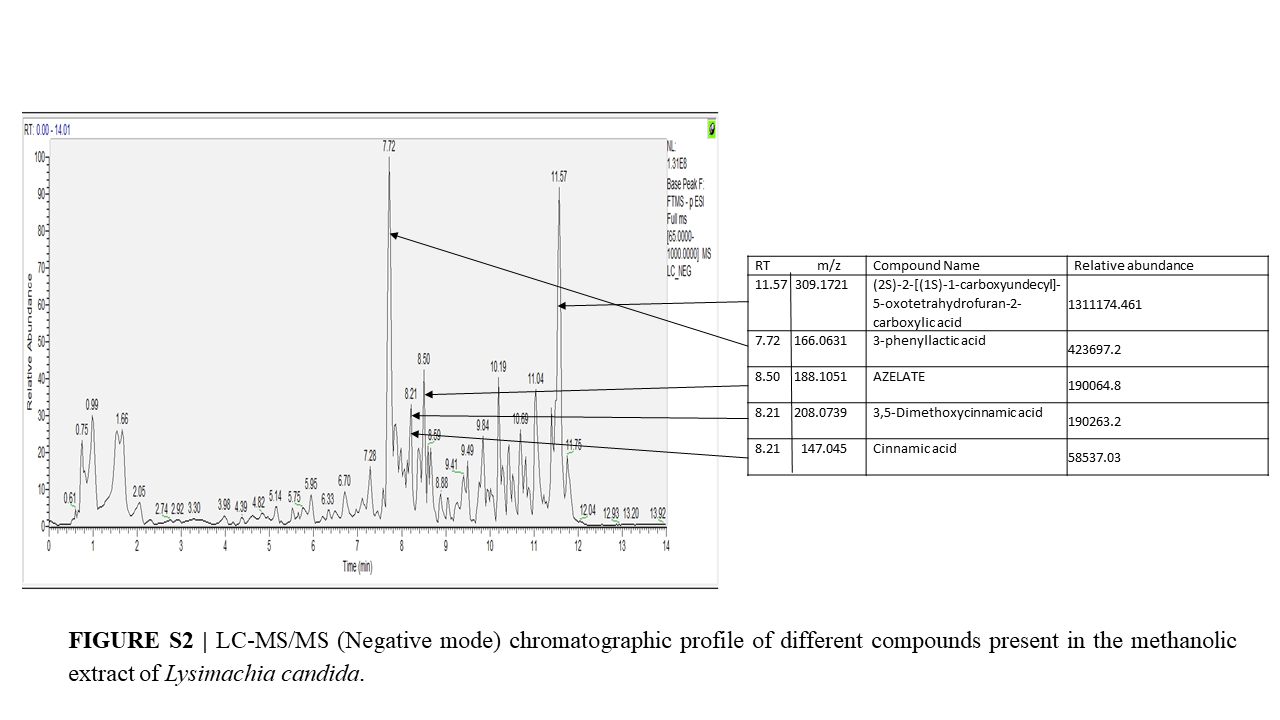

Supplement: Supplementary file 2 [file image2.tif]
