# Supplementary material for: Methanolic Extract of Lysimachia Candida Lindl. Prevents High-Fat High-Fructose-Induced Fatty Liver in Rats: Understanding the Molecular Mechanism Through Untargeted Metabolomics Study
Source: Front Pharmacol. 2021 Apr 15;12:653872. doi: 10.3389/fphar.2021.653872 (PMC8082144; doi:10.3389/fphar.2021.653872)
Supplement: Supplementary file 3 [file image3.pdf]

FIGURE S3A-S3I | MS-MS of major phytoconstituents identified from the plant extract.

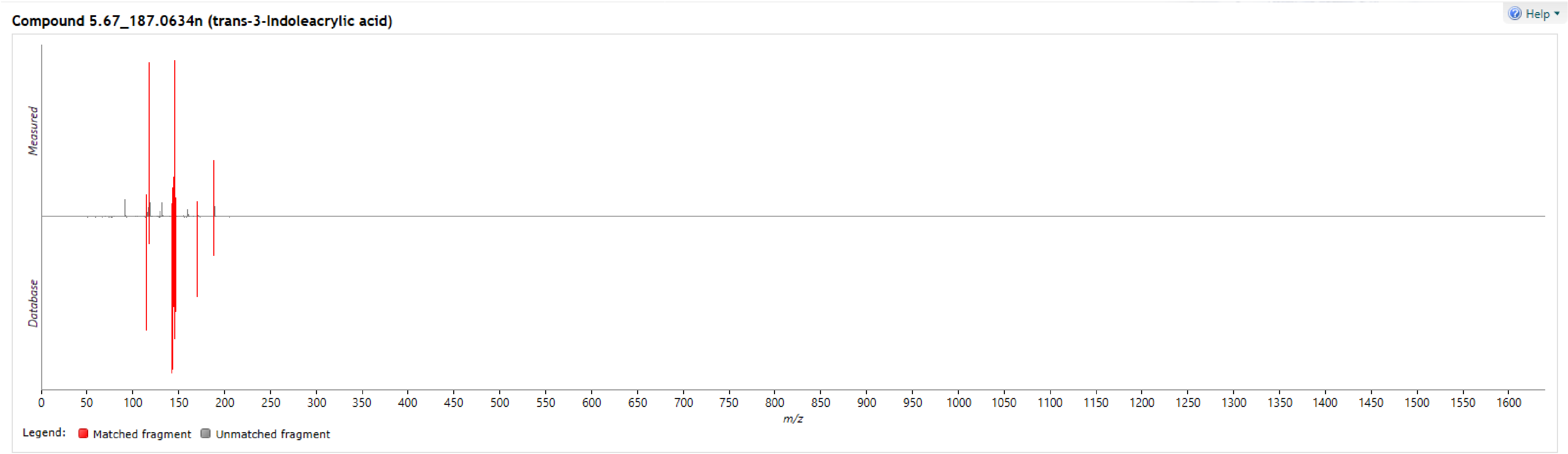

FIGURE S3A | MS-MS of trans-3-Indoleacrylic acid.

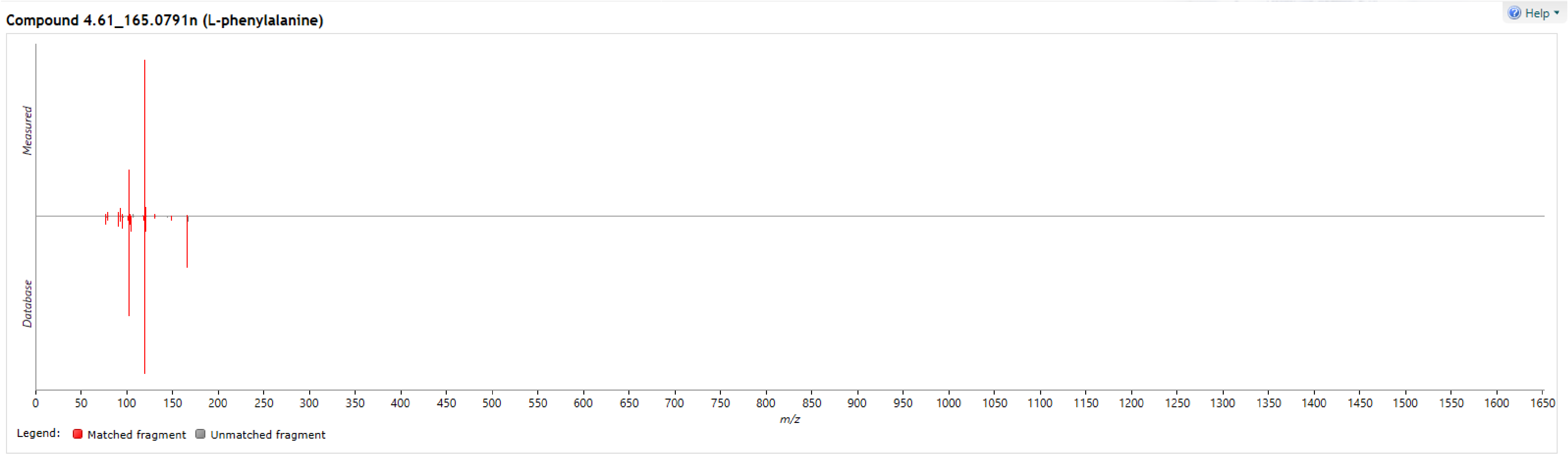

FIGURE S3B | MS-MS of L-phenylalanine.

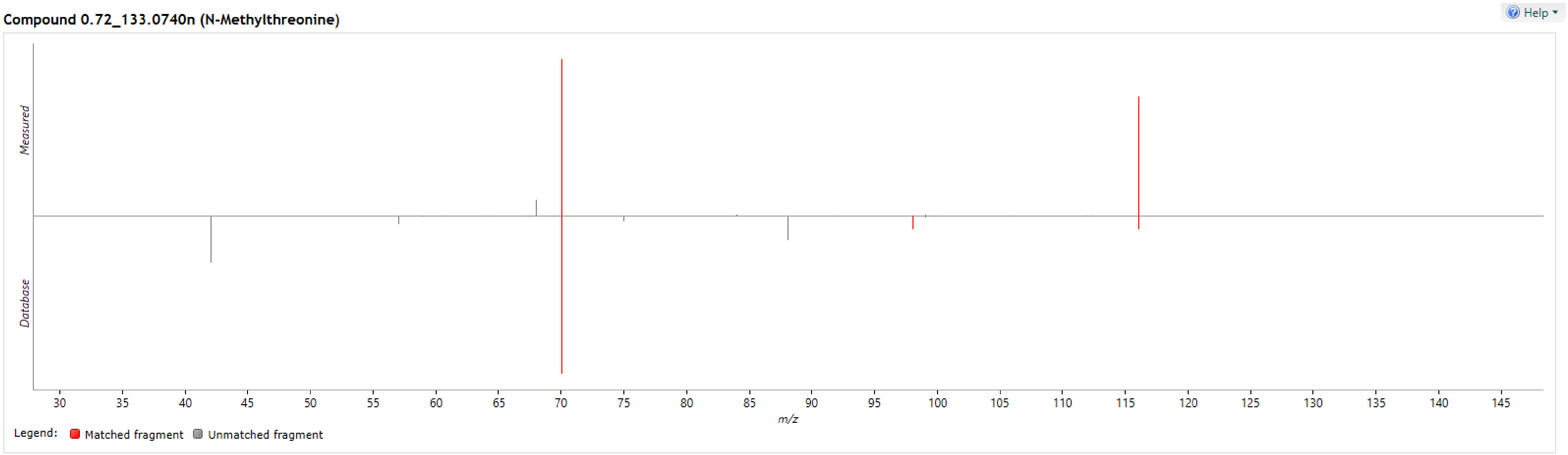

FIGURE S3C | MS-MS of N-Methylthreonine.

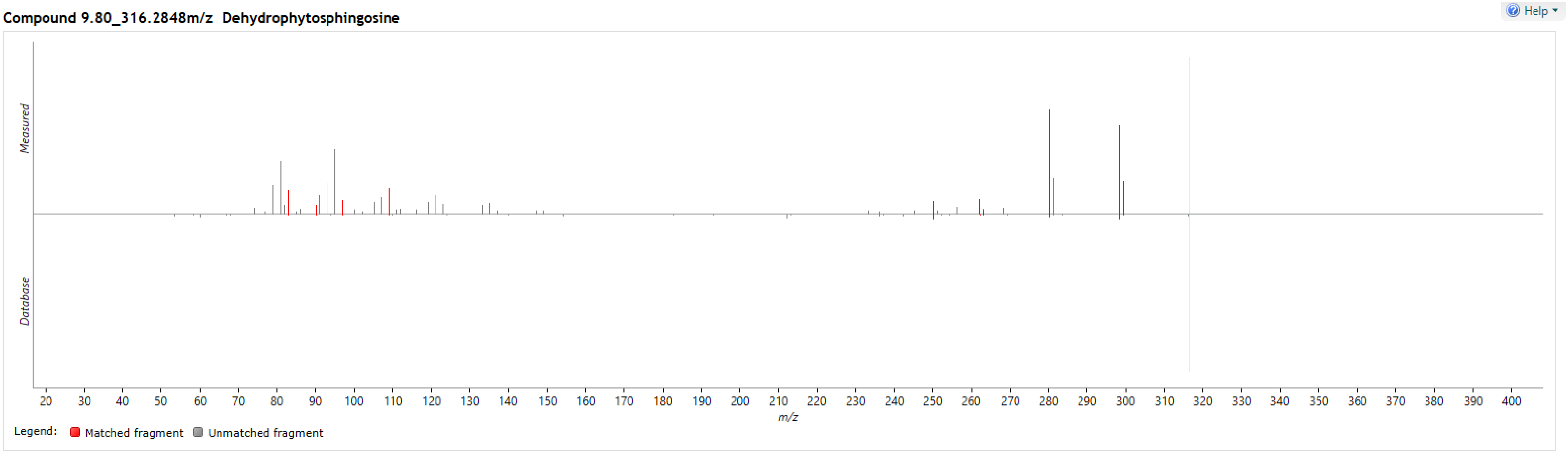

FIGURE S3D | MS-MS of Dehydrophytosphingosine.

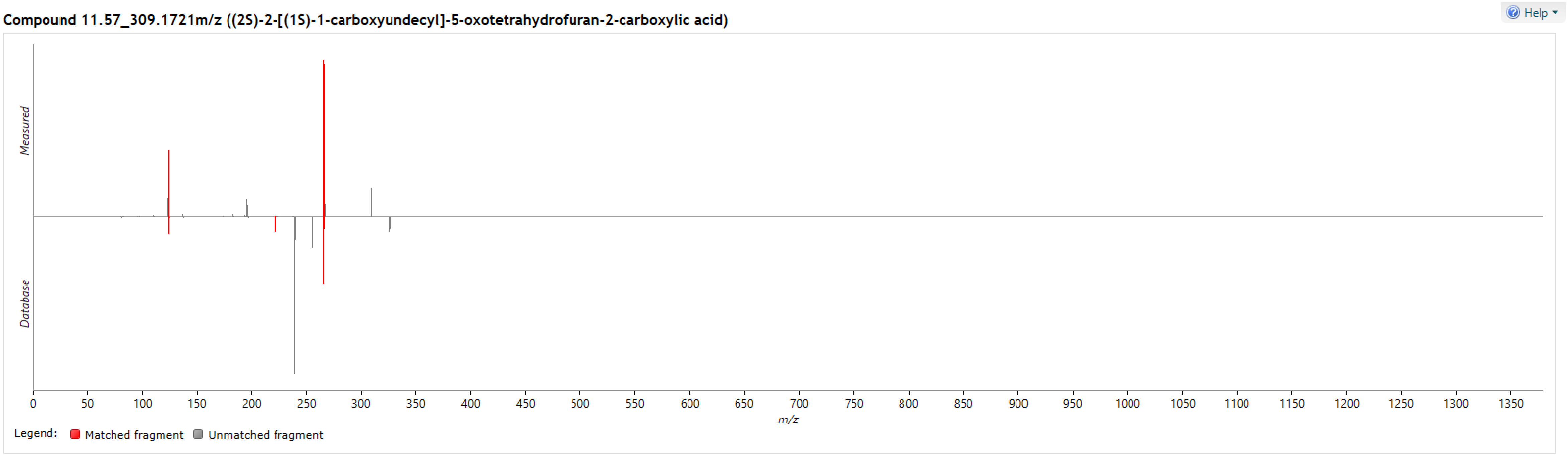

FIGURE S3E | MS-MS of ((25)-2-[(1S)-1-carboxyundecyl)-5-oxotetrahydrofuran-2-carboxylic acid).

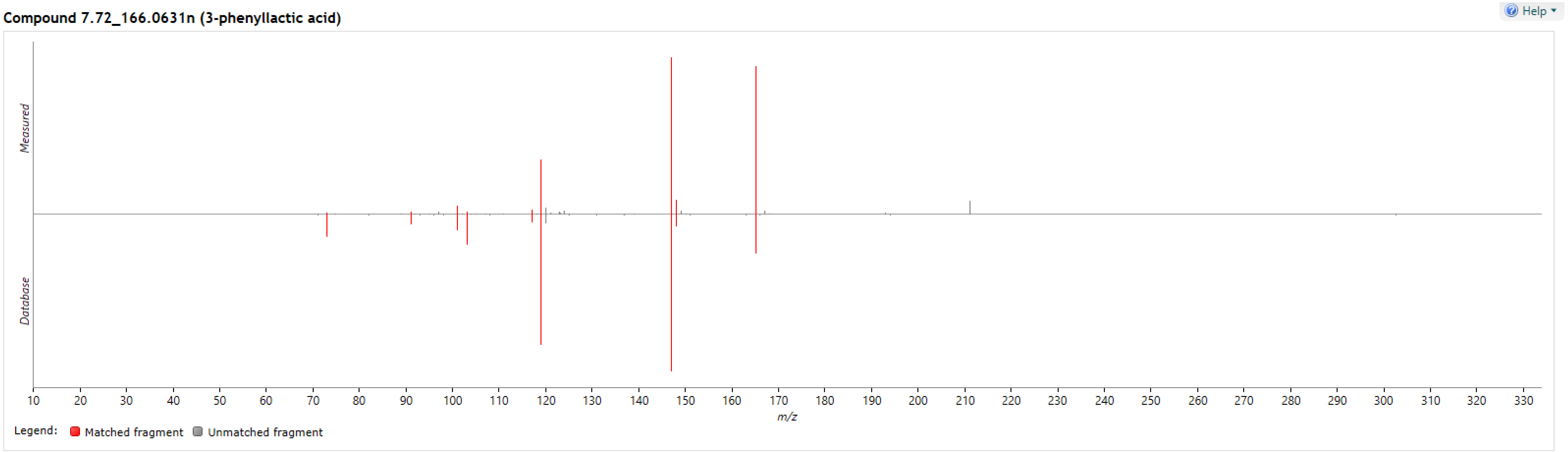

FIGURE S3F | MS-MS of 3-phenyllactic acid.

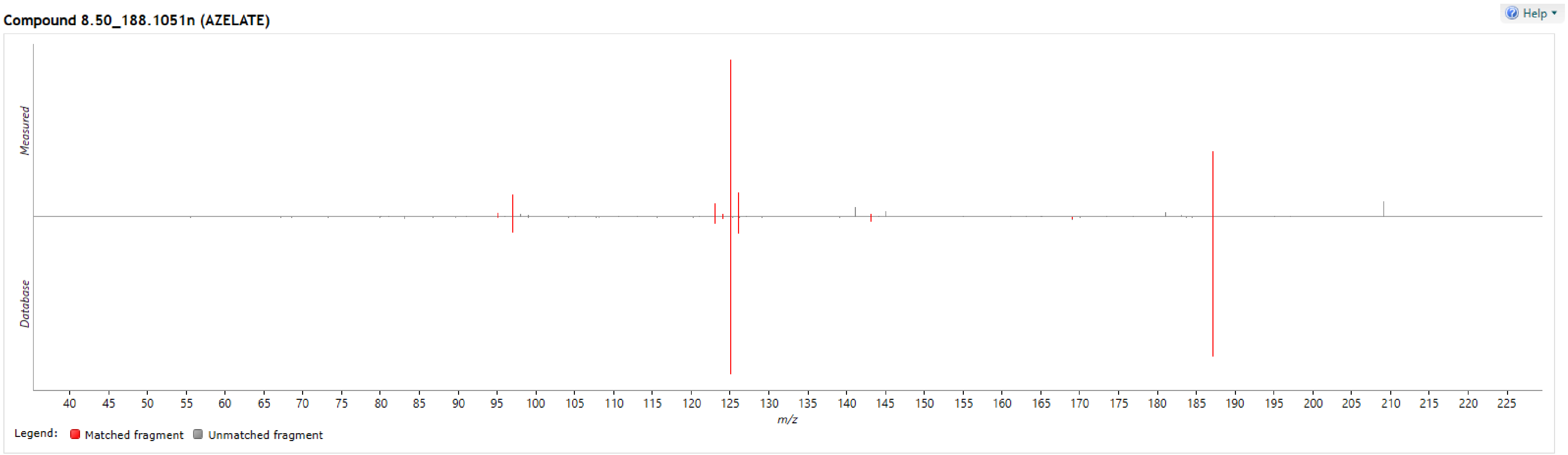

FIGURE S3G | MS-MS of AZELATE.

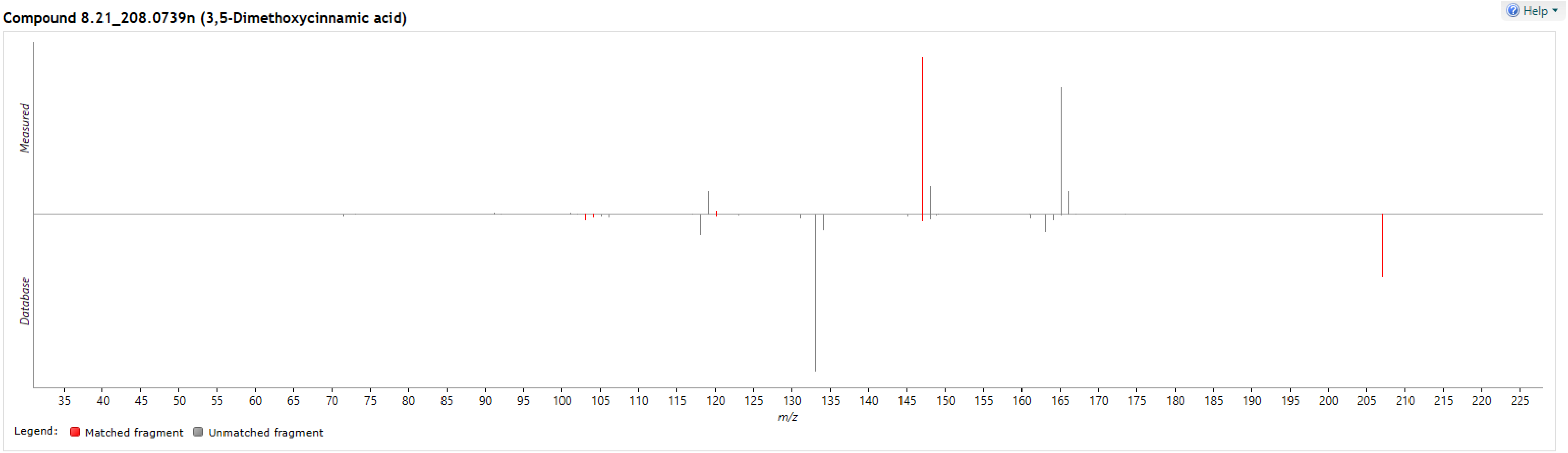

FIGURE S3H | MS-MS of 3,5-Dimethoxycinnamic acid.

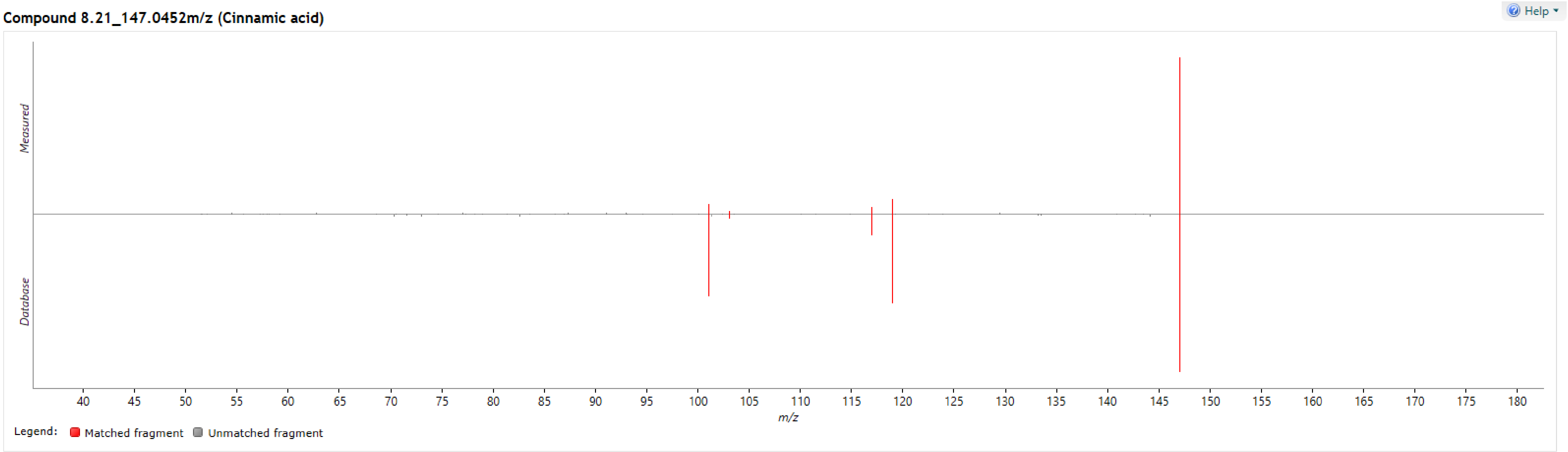

FIGURE S3I | MS-MS of Cinnamic acid.
